# Trihexyphenidyl Alters Its Host’s Metabolism, Neurobehavioral Patterns, and Gut Microbiome Feedback Loop—The Modulating Role of *Anacyclus pyrethrum*

**DOI:** 10.3390/antiox13010026

**Published:** 2023-12-21

**Authors:** Abdelmounaim Baslam, Hajar Azraida, Rachida Aboufatima, Mohamed Ait-El-Mokhtar, Ilham Dilagui, Samia Boussaa, Abderrahman Chait, Marouane Baslam

**Affiliations:** 1 Laboratory of Pharmacology, Neurobiology, Anthropobiology and Environment, Department of Biology, Faculty of Sciences Semlalia, Cadi Ayyad University, Marrakech 40000, Morocco; baslamounaim@gmail.com (A.B.);; 2 Laboratory of Biological Engineering, Faculty of Sciences and Technology, Sultan Moulay Slimane University, Beni Mellal 23000, Morocco; 3 Laboratory of Biochemistry, Environment & Agri-Food URAC 36, Department of Biology, Faculty of Science and Techniques—Mohammedia, Hassan II University of Casablanca, Mohammedia 20000, Morocco; mohamed.aitelmokhtar@gmail.com; 4 Laboratory of Microbiology, University Hospital Mohamed VI, Faculty of Medicine and Pharmacy, Cadi Ayyad University, Marrakech 40000, Morocco; 5 Higher Institute of Nursing and Health Techniques, Ministry of Health and Social Protection, Rabat 10000, Morocco; samiaboussaa@gmail.com; 6 Laboratory of Biochemistry, Department of Applied Biological Chemistry, Faculty of Agriculture, University of Niigata, Niigata 950-2181, Japan; 7 Center of Agrobiotechnology and Bioengineering, Research Unit Labelled CNRST (Centre AgroBiotech-URL-7 CNRST-05), Cadi Ayyad University, Marrakech 40000, Morocco; 8 Laboratory of Agro-Food, Biotechnologies and Valorization of Plant Bioresources (AGROBIOVAL), Department of Biology, Faculty of Science Semlalia, Cadi Ayyad University (UCA), Marrakech 40000, Morocco

**Keywords:** addiction, drug dependence, microbiome, gut–brain axis, pellitory, oxidative stress, substance abuse, trihexyphenidyl (Artane), dependence, *Anacyclus pyrethrum*

## Abstract

Trihexyphenidyl (THP)—a synthetic anticholinergic medication used to manage parkinsonism and extrapyramidal symptoms—has gained significant clinical recognition. However, there is a critical gap in understanding its withdrawal effects. This study investigates the intricate interplay between gut microbiota and oxidative stress during THP withdrawal. Furthermore, it explores the therapeutic potential of *Anacyclus pyrethrum* (AEAP) for alleviating the associated adverse effects. This comprehensive research combines behavioral tests, biochemical analysis, gut microbiome assessment utilizing matrix-assisted laser desorption ionization–time of flight mass spectrometry (MALDI-TOF MS), and oxidative stress measures. The results reveal that the chronic administration of THP leads to severe withdrawal syndrome, marked by heightened anxiety, depressive-like behaviors, increased cortisol levels, elevated oxidative stress, and gut dysbiosis. However, the administration of AEAP alongside THP shows a significant capacity to mitigate these deleterious effects. Co-treatment and post-treatment with AEAP increased bacterial density and diversity, promoting the proliferation of beneficial bacteria associated with improved gut health. Furthermore, AEAP administration reduced cortisol levels and exhibited potent antioxidant properties, effectively countering the THP-induced oxidative damage. This study highlights the withdrawal effects of THP and underscores the therapeutic potential of AEAP for managing these symptoms. The findings reveal its promising effects in alleviating behavioral and biochemical impairments, reducing oxidative stress, and restoring gut microbiota, which could significantly impact the clinical management of THP withdrawal and potentially extend to other substance withdrawal scenarios.

## 1. Introduction

Trihexyphenidyl (THP) is a synthetic anticholinergic medication renowned for its efficacy in managing parkinsonism and extrapyramidal symptoms (EPS) resulting from antipsychotic drug use [1]. While it has well-documented clinical benefits for treating neuroleptic-induced extrapyramidal syndromes in various psychiatric disorders [1], its utilization is also linked with a range of negative effects, such as the diminished therapeutic effect of neuroleptics [2], an alteration in the metabolism of psychotropic drugs, central cholinergic crises, delirium-like states, choreiform and respiratory dyskinesia, memory impairment, and sinus bradycardia [3]. Studies have notably indicated the abuse potential of anticholinergic medications, primarily when prescribed for treating extrapyramidal symptoms (EPS) in psychiatric settings. Among these medications, THP has been recognized as the most potent and frequently abused [4]. The prevalence of THP abuse ranges from 1.1% in the general population to as high as 34% in psychiatric settings [5].

The mood-stimulating and euphoric effects of THP often render it more appealing to patients than antipsychotic medications, potentially leading to habituation or chronic dependence [6]. THP binds to the muscarinic and dopaminergic receptors to mitigate extrapyramidal side effects. At high doses, its affinity for dopaminergic receptors is likely responsible for its neuropsychiatric effects (i.e., hallucinations and euphoria), contributing to its potential abuse by stimulating the reward system of the central nervous system (CNS) [7]. Mahal et al. observed a range of withdrawal symptoms upon discontinuation of antiparkinsonian drugs, including anxiety, depression, motor agitation, hallucinations, gastrointestinal distress, physical complaints, and increased psychopathology [8]. Anticholinergics operate by competitively inhibiting acetylcholine at the muscarinic receptors and, at very high doses, they may partially block nicotinic receptors, including those at the neuromuscular junction [9]. Ongoing research is vital for developing innovative approaches to prevent, predict, and treat substance abuse at various stages. This is particularly significant as the development of viable treatment strategies capable of addressing substance use disorders (SUDs) and withdrawal symptoms remains a significant challenge for clinical practice. Many individuals with SUDs experience a relapse even after extended periods of abstinence, underscoring the urgent need for effective interventions to mitigate the detrimental effects of substance abuse on the brain [10]. 

The microbiota–gut–brain (MGB) axis describes the dynamic interaction between the CNS and the gut microbiota (GM), involving pathways that influence neurotransmission and behavior [11,12,13]. Recent research has shed light on the intricate interplay between oxidative stress and the GM, uncovering how the communication between an imbalance in redox processes and gut dysbiosis can exert a significant impact on the MGB [14,15]. The concept of the MGB axis suggests a dynamic interaction, where the microbiota residing in the intestinal lumen can exert an influence over the CNS activity of its host, affecting both vegetative and cognitive functions. Conversely, brain activity can, in turn, shape the development and composition of the GM [16]. Moreover, emerging evidence suggests that gut dysbiosis may contribute to oxidative stress imbalances within the CNS, potentially resulting in increased levels of oxidative stress in the brain [17]. These reciprocal interactions have the potential to make a substantial impact on global health and to offer innovative avenues for preventive and therapeutic interventions. 

Nowadays, aromatic and medicinal plants are no longer limited to remedies for disadvantaged communities in developing countries; they have become valuable sources of bioactive molecules highly sought after by the pharmaceutical, agri-food, cosmetic, and perfumery industries [18]. Aromatic and medicinal plants are broadly utilized in both modern and traditional medicine [19], with ca. 50% of the medications prescribed by doctors originating from natural sources [20]. *Anacyclus pyrethrum*, the pellitory, is renowned for its pharmacological properties, which encompass antinociceptive, antioxidant, antidepressant, and anticonvulsive qualities [21,22]. Notably, it has exhibited promise in addressing SUDs during the challenging phases of cigarette smoke and ecstasy withdrawal [23,24]. This research suggests that *A. pyrethrum* may act as a valuable agent for modulating the GM, thereby indicating its potential to leverage the brain–gut connection to alleviate SUD-related complications and facilitate recovery.

This study, to the best of our knowledge, represents the first examination of THP withdrawal effects. We aimed to examine the uncharted relationship between the GM and oxidative stress during THP withdrawal, and to investigate the therapeutic potential of *A. pyrethrum* in mitigating the associated adverse effects.

## 2. Material and Methods

### 2.1. Animals 

Male Sprague Dawley rats, with a weight of 190 ± 15 g, were individually placed in clear cages within a regulated environment. The temperature was kept constant at 22 ± 2 °C, and the humidity was maintained at 50 ± 10%. A light/dark cycle of 12 h each was adhered to. The rats had unrestricted access to both water and food, which were provided ad libitum. Before the commencement of the experiments, the rats were acclimatized to the laboratory environment for a period of seven days. All procedures involving animals were conducted following the guidelines outlined by the European Council Directive for Care and Use of Laboratory Animals (EU2010/63). Prior to commencement, the Institutional Local Review Board approved the study protocol for animal experimentation, with the protocol code BS987/03/23 and an approval date of March 2023. 

For this study, the rats were divided into four groups, each containing six animals:

(1) The control group received a vehicle (saline solution 0.9%).
(2) The THP-dependent group underwent 30 days of daily THP administration (from day 4 to day 33 of the experiment), followed by a 7-day withdrawal phase (from day 34 to day 40).
(3) The THP + post-AEAP group included rats treated with a daily THP administration for 30 days (from day 4 to day 33 of the experiment), and post-treated with AEAP (200 mg/kg) for 7 days (from day 34 to day 40), to assess the potential curative effects of AEAP.
(4) The THP + co-AEAP group involved rats administered AEAP (200 mg/kg) 30 min prior to THP administration for 30 days (from day 4 to day 33), followed by a 7-day withdrawal phase (from day 34 to day 40), to explore the potential preventive effects of AEAP (Figure S1).

Before the main study on the effects of AEAP on THP withdrawal, a preliminary investigation was carried out to evaluate the pharmacological effects of different AEAP doses (100, 200, 400, and 800 mg/kg) on anxiety and depression. The results identified the 200 mg/kg dose as the most effective and, thus, was selected for this study.

### 2.2. Drugs Administration

Commercially obtained THP (Artane, MAPHAR, Casablanca, Morocco), dissolved in saline solution, was administered in a progressively increasing dose (20% increase/week), starting with 5 mg/kg/day and reaching 10 mg/kg/day through daily intraperitoneal injections for 30 days, emulating the progressive tolerance seen in human addiction [3,25]. 

### 2.3. Plant Material and Preparation of the Aqueous Extract of A. pyrethrum

The roots of *A. pyrethrum* were gathered from their natural habitat in the Bin El Ouidan region of Morocco (32°7′48″ latitude N/6°27′36″ longitude W). Their authentication was initially conducted by botanist Professor A. Ouhammou and pharmacologist Professor A. Chait of the Faculty of Science Semlalia, Cadi Ayyad University. Subsequently, the plants were stored under voucher specimen MARK-1003 in the herbarium of the Department of Biology, Faculty of Science Semlalia, Cadi Ayyad University, Marrakesh, Morocco. The extraction process followed established procedures [23]. Dried root powder was obtained by crushing the roots, which were then subjected to extraction with distilled water (1 g/10 mL) under agitation for 24 h. The resulting aqueous macerate underwent centrifugation (1200 rpm), filtration, lyophilization using a Christ instrument, and was subsequently stored in amber bottles at 4 °C until utilized. 

The toxicity assessment of AEAP at doses of 1000, 2000, and 5000 mg/kg confirmed its safety. During the 14-day administration of AEAP, there were no instances of mortality, and no notable alterations in body or organ weights were detected (*p* > 0.05). The Lethal Dose 50 (LD_50_) for AEAP was found to be above 5000 mg/kg, indicating its non-toxic characteristics.

### 2.4. Conditioned Place Preference (CPP)

The THP-induced conditioned place preference (CPP) test was conducted following a previously established protocol [23]. The CPP apparatus consisted of three Polyvinyl Chloride (PVC) compartments: two larger conditioning side chambers (30 cm × 25 cm × 30 cm) with distinct somatosensory cues, such as colored walls (white or zebra) and different floor surfaces, and a middle neutral chamber (11 cm × 25 cm × 30 cm). The CPP procedure consisted of three phases: preconditioning, conditioning, and post-conditioning (dependence). During the preconditioning (day 1–3), the rats were put in the middle chamber with the doors opened, allowing them unrestricted access to both compartments. Their behavior and preferences were observed and recorded for 15 min to establish the baseline preference for each chamber. Rats showing a clear preference for one side compartment over the other were excluded from this study. 

In the second phase (conditioning: days 4–9), the rats received alternating injections of either THP or saline solution two times per day: in the morning (10:00 a.m.) and evening (8:00 p.m.) for six days. After receiving THP, the rats were confined to the zebra compartment for 45 min, while after saline injection, they were confined to the white compartment. The control group received saline injections during the rotated sessions throughout the conditioning and post-conditioning phases. On day 9, the rats underwent re-testing for THP-CPP. They were given free access to both the white and zebra chambers for 15 min, and their behavior was monitored using a camera connected to a computer interface. The number of entries to the THP-paired chamber and the total entries were recorded to calculate the CPP score. The time spent in each compartment was also measured. 

During the post-conditioning phase (days 10–33), the rats received daily injections of either THP, for group 2, or THP + co-AEAP, for group 4. Following the injections, they were confined to the zebra compartment for 45 min. At the end of the withdrawal phase (day 40), the rats were allowed unrestricted entry to all the apparatus, and their behavior was observed for 15 min.

### 2.5. Behavioral Assessment

#### 2.5.1. Open Field Test (OFT)

To assess exploratory behavior and locomotor activity, the Open Field Test (OFT) was conducted in a brightly lit room. The animals were individually placed in the center of a white arena measuring 80 cm × 80 cm × 40 cm. The arena was divided into 25 equal squares. The rats were given 10 min to freely explore the unfamiliar environment. During the observation period, the number of squares crossed by the rats using all four legs and the number of instances where the animals stood on their hind legs (rearing behavior) to explore their environment were recorded [26]. After each test, the OFT apparatus was thoroughly cleaned using a 10% ethanol solution to eliminate any potential olfactory cues that could influence subsequent testing sessions.

#### 2.5.2. Porsolt’s Forced Swim Test (FST)

The Forced Swim Test (FST) is a widely employed behavioral test for evaluating depression-like behavior in rats [27]. Each rat was individually positioned in a transparent cylinder with dimensions of 21 cm in diameter and 60 cm in height. The cylinder was filled with water to a level of 40 cm and maintained at a temperature of 25 ± 1 °C. The duration of immobility was subsequently recorded over a 10 min period. Immobility was characterized as the duration for which the rats displayed minimal movement in the water, demonstrating an inactive attitude, like limited swimming, diving, and jumping, with the primary objective of keeping their heads above water. An elevation in immobility time signified the presence of a depressive-like effect in the rats’ behavioral patterns.

#### 2.5.3. Elevated plus Maze (EPM)

To assess anxiety-like behaviors, the Elevated Plus Maze (EPM) test was conducted. The apparatus was positioned in a separate room at a height of 100 cm above the floor. The maze consisted of two open arms and two enclosed arms, each measuring 50 cm × 10 cm. The enclosed arms were equipped with walls measuring 40 cm in height, while the open arms were wall-less. The EPM also featured a central zone measuring 10 cm × 10 cm.

At the beginning of each test, the rat was positioned in the central zone, facing the intersection of the maze, and their exploratory behavior was observed for a duration of 10 min. The number of entries and the time spent in both the open and closed arms (defined as having all four legs in the arm) were recorded as the dependent measures [28]. After each test, the EPM apparatus was cleaned using a 10% ethanol solution to minimize the introduction of pheromonal cues. Tukey’s *t* tests were employed to identify significant differences in open/closed arm time or entries. All behavioral tests were carried out on both day 3 and day 40 during the preconditioning and withdrawal phases, respectively. These tests were performed during the light phase of the light/dark cycle.

### 2.6. Biochemical Analyses

After conducting the behavioral assessments on the 40th day of the withdrawal phase, the animals were euthanized under ether anesthesia. Blood samples were obtained using chilled centrifuge tubes without anticoagulants, allowed to clot at 25 °C for 30 min, and then centrifuged at 1500× *g* for 15 min to obtain the serum. The obtained serum was stored at −20 °C for cortisol measurement using a biochemical machine (Cobas 6000, Roche, Basel, Switzerland).

### 2.7. Oxidative Stress

The rats’ brains were softly removed and placed on a cold surface to maintain tissue integrity. The hippocampus, the focus of this study, was dissected using precise anatomical landmarks and standardized coordinates based on a brain atlas [29]. These hippocampus tissues were homogenized in 20 mM Tris-HCl buffer (pH 7.4)—to maintain the stability of the extracted molecules—on ice to break down the tissue into smaller fragments and to ensure uniformity for the subsequent biochemical analysis. 

#### 2.7.1. LPO Assay

The assessment of lipid peroxidation levels involved quantifying the thiobarbituric acid-reacting substances (TBARS) in the hippocampus homogenates, following an established protocol [30]. In brief, a segment of crude homogenate (100 mg) underwent centrifugation at 4 °C (1000× *g* for 10 min), and the resulting supernatant was mixed with 1 mL of 10% trichloroacetic acid (TCA) and 1 mL of 0.67% thiobarbituric acid (TBA). The mixture was then heated in a boiling water bath for 15 min, and butanol (2:1, *v*/*v*) was added to the solution. Following centrifugation (1500 rpm for 10 min), the absorbance was measured at 535 nm to determine the TBARS levels, expressed as nmol of malondialdehyde (MDA) per gram of wet tissue [31].

#### 2.7.2. Catalase (CAT) Activity

CAT activity was determined by assessing the production of H_2_O and O_2_ using an H_2_O_2_-dependent method [32]. Briefly, samples (0.05 mL) were combined with 1 mL H_2_O_2_ (0.019 M) and 1.95 mL 50 mM phosphate buffer in a 3 mL quartz cuvette. Absorbance was recorded at 240 nm at time 0 (T0) and every 30 s for 2 min. CAT activity was calculated as mmol of H_2_O_2_/min/mg protein.

#### 2.7.3. Superoxide Dismutase (SOD) Activity

SOD activity was determined by evaluating its ability to inhibit the photoreduction of nitro blue tetrazolium (NBT) using spectrophotometric methods [33]. The assay systems were prepared by combining 2.4 × 10^−6^ M riboflavin, 0.01 M methionine, 1.67 × 10^−4^ M NBT, and 0.05 M potassium phosphate buffer (pH 7.4). Absorbance was measured at 560 nm, and one unit of SOD activity was defined as the amount of enzyme protein causing a 50% reduction in the rate of NBT reduction.

### 2.8. Gut Microbiota Determination 

Following the behavioral tests, 1.5 cm segments of the intestinal tract were aseptically collected at the time of euthanasia from the respective groups to assess the impact of THP and AEAP on the bacterial density and relative bacterial abundance. These intestinal samples were immediately placed in sterile tubes. Subsequently, each sample was evenly spread on the surface of a sterile, dry nutrient agar medium within Petri dishes. The samples were then incubated at 37 °C for 72 h under aerobic and anaerobic conditions [23]. After incubation, the bacterial strains were meticulously enumerated, and their classification was determined using matrix-assisted laser desorption ionization—time of flight mass spectrometry (MALDI-TOF MS).

#### 2.8.1. MALDI-TOF MS Spectra for Mass Spectral Profiles

To enhance microbe extraction before applying the matrix solution to the bacterial spots, a preliminary procedure utilizing formic acid (FA) was implemented. This step capitalized on the matrix’s acidic pH to enhance the extraction of ribosomal proteins. Initially, the sample was mixed with 300 μL of high-pressure liquid chromatography (HPLC)-grade water, followed by the addition of 900 μL of 100% ethanol. The resulting mixture underwent centrifugation at 15,000× *g* for 2 min to isolate the bacterial pellet. Subsequently, this pellet was dried and reconstituted in 50 μL of FA (70% in water). After vortexing the mixture, 50 μL of acetonitrile (ACN, Sigma, Schnelldorf, Germany) was added, followed by another round of centrifugation at 15,000× *g* for 2 min.

A 1 μL aliquot of bacterial extract supernatant was dispensed in duplicate onto a polished MSP 96-spot steel plate (Bruker-Daltonics, Billerica, MA, USA) and allowed to dry at room temperature. For proper instrument calibration during data acquisition and processing, a bacterial test standard (1 μL) from Bruker-Daltonics was pipetted onto two MALDI target spots. Following this, the bacterial samples were covered with α-cyano-4-hydroxycinnamic acid (1 μL) from Sigma Aldrich, St. Louis, MO, USA, and allowed to air-dry. They were then reconstituted in a mixture of 1 μL consisting of 70% FA and ACN before undergoing analysis using MALDI-TOF MS. Calibration of the instrument before each data acquisition session was carried out using the bacterial test standard (BTS).

#### 2.8.2. MALDI-TOF MS Data Acquisition and Processing

The protein mass spectra from the samples were captured using the reference database V.3.1.2.0 (3995 entries) and the research-use-only (RUO) MALDI Biotyper software (version 3.0) provided by Bruker-Daltonics. This analysis utilized laser frequencies of 20 or 60 Hz in linear and positive modes, encompassing a mass range of 2000–20,000 Da. Standard operational parameters comprised an ion source voltage of 18.25 kV, acceleration at 20 KV, and pulse ion extraction at 370 ns. Each strain was represented by thirty individual spectra obtained from four independent cultures, each with three technical replicates. The results were interpreted in accordance with the manufacturer’s guidelines for MALDI-TOF MS analysis. A score of 2.0 indicated species-level identification with high confidence, scores ranging from 1.700 to 1.999 signified genus-level identification, and scores below 1.7 were considered unidentified.

### 2.9. Histological Study 

Following the completion of the behavioral assessments and blood sample collection, the intestines were dissected and immersed in a 10% formalin solution for an overnight fixation. Subsequently, the specimens underwent a dehydration process involving a series of graded alcohol solutions before being embedded in paraffin wax. Sections, measuring 4 μm in thickness, were prepared and stained with hematoxylin and eosin for a pathological analysis, following the protocol described by Malatesta [34]. 

### 2.10. Statistical Analyses 

The data were analyzed and are presented as the mean ± SEM (standard error of the mean) with a sample size of *n* = 6. Group differences were assessed using a two-way analysis of variance (ANOVA), followed by a Tukey’s post hoc test, when *p* < 0.05. Statistical significance was considered for a *p*-value less than 0.05. The statistical analysis was conducted using Graphpad Prism 09 (San Diego, CA, USA).

## 3. Results

### 3.1. Trihexyphenidyl-Induced CPP 

**Figure 1:**
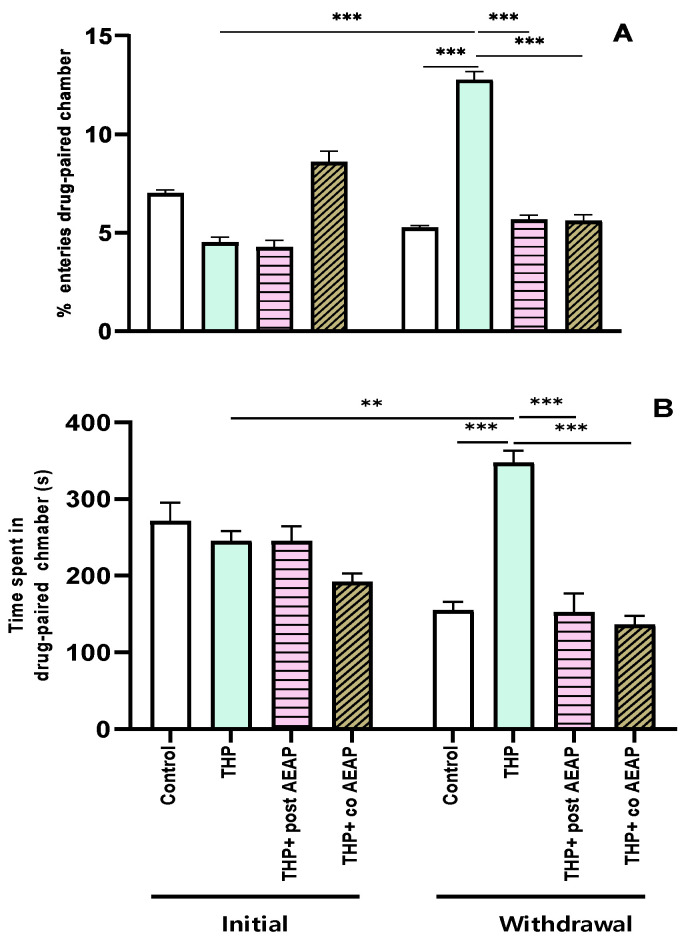
Effects of trihexyphenidyl and/or aqueous extract of *Anacyclus pyrethrum* (AEAP) on dependence of conditioned place preference (CPP): (**A**) % of entries and (**B**) time spent in the drug-paired chamber during the CPP test. Data are mean ± SEM (*n* = 6 per group). Data were analyzed using one-way ANOVA, followed by Tukey’s post hoc test. ** *p* < 0.01; *** *p* < 0.001.

The preference for THP was assessed using the conditioned place preference test, revealing significant differences among the groups and phases. The analysis showed statistical significance for both the percentage of entries (F_3,32_ = 111.2; *p* < 0.001) and the time spent in the drug-paired chamber (F_3,32_ = 16.32; *p* < 0.001). During the withdrawal phase, the groups previously exposed to THP exhibited a substantial increase (4.75 ± 0.27 during the initial phase and 12.75 ± 0.45 during withdrawal) in entries and time spent in the drug-paired chamber compared to the initial phase and the control group during withdrawal (*p* < 0.001) (Figure 1A,B). Notably, the CPP was not induced in the AEAP-treated groups.

### 3.2. Trihexyphenidyl Withdrawal-Induced Anxiety and Depression, and the Potential of AEAP to Alleviate Adverse Outcomes

**Figure 2:**
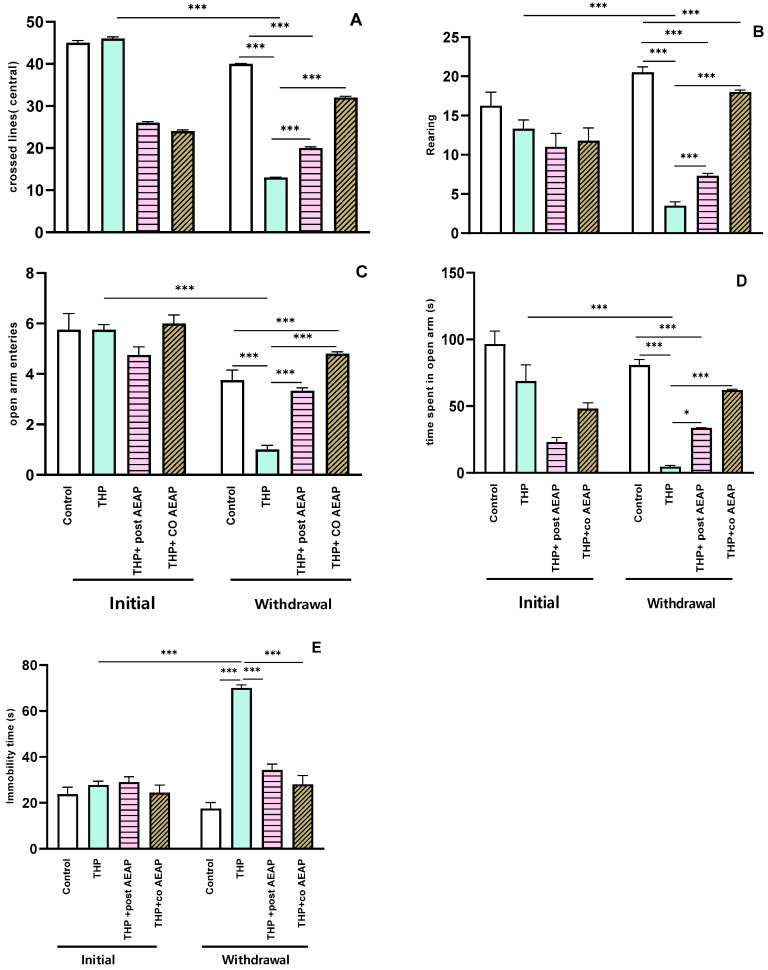
Effects of trihexyphenidyl and/or aqueous extract of *A. pyrethrum* (AEAP) on (**A**) the central crossed lines, (**B**) the number of rearings in the OFT, (**C**) the open arm entries, (**D**) the time spent in the open arms in the EPM, and (**E**) the immobility time in the FST. Data represent mean ± SEM (*n* = 6 per group). The data presented reflect the mean values along with the standard error of the mean (SEM), with a sample size of 6 per group. Statistical analyses for each phase (initial or withdrawal) were conducted using a one-way ANOVA followed by Tukey’s test. Significance levels are denoted as * *p* < 0.05, and *** *p* < 0.001.

Anxiety-like behaviors were evaluated using the OFT and EPM. The results reveal that the rats experiencing THP withdrawal exhibited anxiety-like symptoms, as evidenced by a notable reduction in the number of central lines crossed (Figure 2A) and the number of rearings (Figure 2B). Additionally, there was a marked reduction in the number of entries into the open arms (Figure 2C) and the time spent (Figure 2D) in the EPM. This signifies heightened anxiety during withdrawal, statistically significant both when compared to the initial phase (*p* < 0.001) and to the control group during withdrawal (*p* < 0.001). Concerning depressive-like behaviors, as assessed using the Forced Swim Test, the immobility time significantly decreased in the THP-treated groups compared to the other groups (*p* < 0.001) (Figure 2E). In contrast, during withdrawal, the groups treated with AEAP exhibited an ability to mitigate anxiety and depressive-like behaviors (Figure 2A–E). While the AEAP-treated group showed a significant difference compared to the THP-treated group during withdrawal, no significant difference was observed when compared to the controls, thus indicating both its preventive and curative potential (Figure 2A–E).

### 3.3. Trihexyphenidyl Withdrawal-Induced Cortisol Levels Elevation 

**Figure 3:**
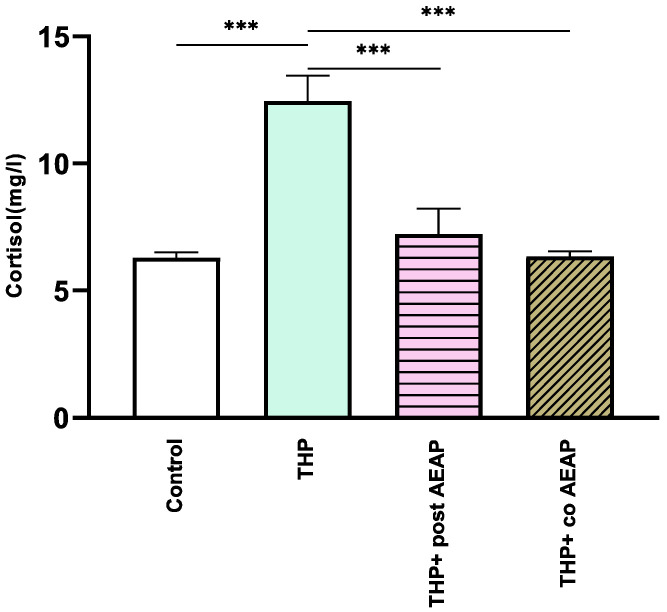
Effects of trihexyphenidyl and/or aqueous extract of *A. pyrethrum* (AEAP) on cortisol levels from the biochemical analyses of the withdrawal phase. The data illustrate the mean value along with the standard error of the mean (SEM), with a sample size of 6 per group. Statistical analysis involved a one-way ANOVA followed by Tukey’s test. Significance levels are indicated as *** *p* < 0.001.

The withdrawal phase involved the assessment of cortisol levels, which serve as a biochemical indicator of stress (Figure 3). The results indicate a notable increase in cortisol levels in the THP-withdrawn group (12.46 mg/L ± 0.46) compared to the vehicle group (6.3 mg/L ± 0.2) (*p* < 0.001). In contrast, no notable differences were observed in the AEAP-treated groups when compared to the controls. Significantly divergent results were observed in comparison to the THP-treated group (F_3,16_ =16.73; *p* < 0.001).

### 3.4. Trihexyphenidyl Withdrawal-Induced Oxidative Stress, and the Antioxidant Capacity of AEAP

**Figure 4:**
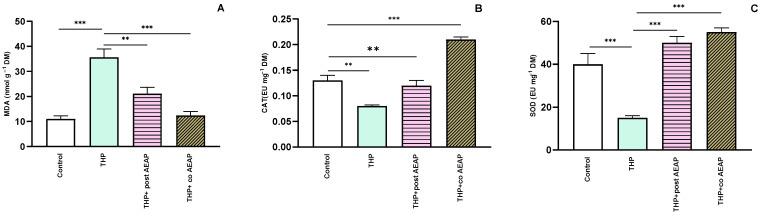
Effects of trihexyphenidyl and/or aqueous extract of *A. pyrethrum* (AEAP) on oxidative stress: (**A**) MDA, (**B**) CAT, and (**C**) SOD values for the withdrawal stage. The provided data depict the mean along with the standard error of the mean (SEM) for each group, with a sample size of 6 per group. Statistical analysis was performed using a one-way ANOVA followed by Tukey’s test. Significance levels are denoted as ** *p* < 0.01, and *** *p* < 0.001.

Next, we assessed the oxidative stress markers, including MDA, CAT, and SOD. The THP-withdrawal group exhibited a notable elevation in MDA levels (35.61 nmol g^−1^ FM ± 3.43) compared to the control group (11.01 nmol g^−1^ FM ± 1.20) (Figure 4A). However, the groups treated with AEAP displayed a significant reduction (12.38 nmol g^−1^ FM ± 1.61) in MDA levels compared to the THP-treated group (F_3,16_ = 23.27; *p* < 0.001) (Figure 4A). For the CAT and SOD levels, the THP group showed decreased levels: 0.08 EU g^−1^ FM ± 0.002 and 15 EU g^−1^ FM ± 1, compared to 0.12 EU g^−1^ DM ± 0.01 and 40 EU g^−1^ FM ± 5 for the control group, respectively (Figure 4B,C). In contrast, the AEAP-treated group displayed a potential to enhance these markers (Figure 4A–C).

### 3.5. Alterations in Microbiota Density and Composition during Trihexyphenidyl Withdrawal, and the Ameliorative Effect of AEAP

**Figure 5:**
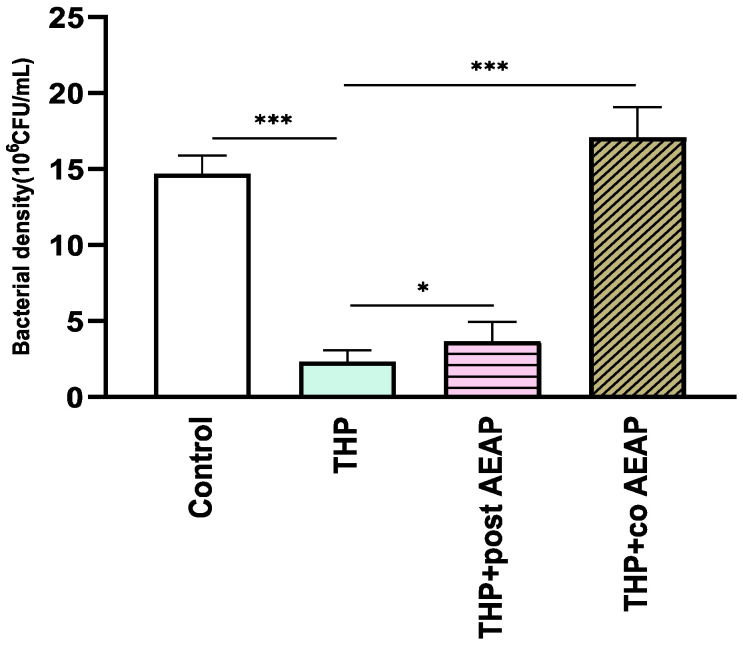
Effects of trihexyphenidyl and/or aqueous extract of *A. pyrethrum* (AEAP) on bacterial density of the gut microbiota during the withdrawal phase. The density represents the total effective bacteria in the sample. Data represent mean ± SEM (*n* = 6 per group). Data were analyzed using a one-way ANOVA followed by Tukey’s test. * *p* < 0.05; *** *p* < 0.001.

The bacterial density significantly varied among the groups (F_3,16_ =165.6; *p* < 0.001) (Figure 5). The THP-withdrawn groups exhibited the lowest bacterial density (2.30 × 10^6^ CFU/mL), which was statisticaly significant compared to the control group (14.70 × 10^6^ CFU/mL, *p* < 0.001). Both groups co- or post-treated with AEAP had a relative abundance of bacterial density, with levels higher than those of the THP groups, at 3.66 × 10^6^ and 17 × 10^6^ CFU/mL, respectively (Figure 5).

**Figure 6:**
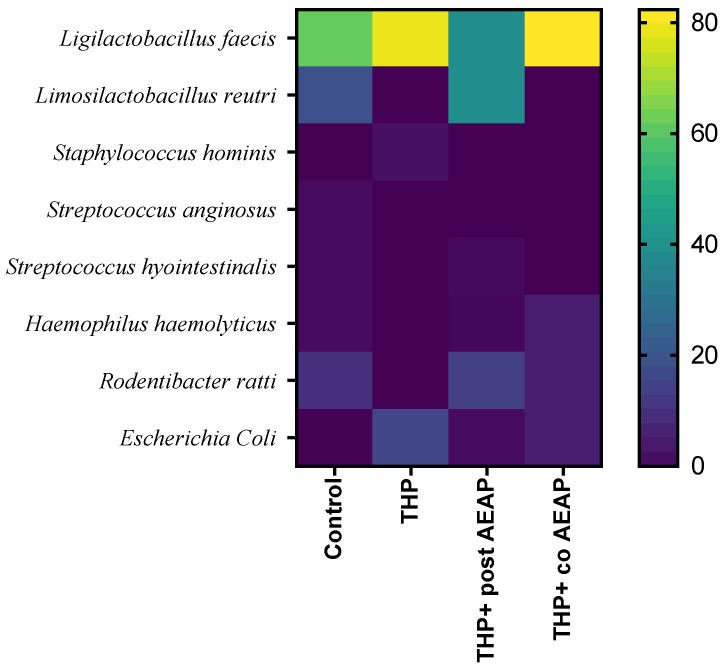
Community heatmap showing microbiota abundance at species level as a percentage of total bacterial sequences. The columns correspond to the groups. The rows correspond to the microbiota at species level.

A MALDI TOF MS analysis was employed for the precise identification of distinct bacterial strains, revealing their predominant classification within the Firmicutes and Proteobacteria phyla. In fact, the Firmicutes phylum was predominantly represented, exhibiting nearly complete dominance (Figure 6). Within the Firmicutes phylum, the orders Bacillales and Lactobacillales were identified, while the Proteobacteria phylum was characterized by the presence of the Pasteurellales and Enterobacterales orders. Notably, the Lactobacillales and Pasteurellacea families emerged as the most prominently represented. A significant perturbation was observed within the Firmicutes and Proteobacteria phyla in the rats withdrawn from THP, suggesting a potential state of gut dysbiosis (Figure 6). In contrast, the co-treated group (THP + AEAP) exhibited an augmented abundance, indicating a potentially ameliorative influence (Supplementary Table S1).

### 3.6. Alterations in Intestinal Tissue during Trihexyphenidyl Withdrawal, and the Ameliorative Effect of AEAP 

**Figure 7:**
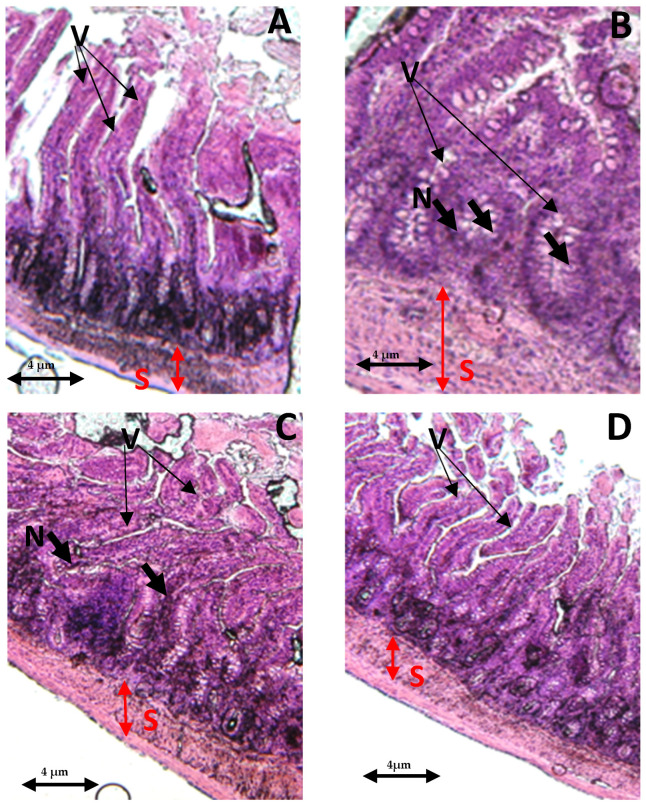
Effects of trihexyphenidyl and/or aqueous extract of *A. pyrethrum* (AEAP) on the histopathological tissues in the small intestines. Samples were examined using an optical microscope (×200) for the following groups: (**A**) the control group, (**B**) the trihexyphenidyl-withdrawn group, (**C**) the trihexyphenidyl + post-AEAP group, and (**D**) the trihexyphenidyl + co- AEAP. Notable histopathological changes were identified, including alterations in villi structure (V), submucosal layer (S), and the presence of large fissures indicative of villus necrosis (N). Scale bar = 4 μm.

In the control group, both the villi and glands had a normal appearance, with no signs of an inflammatory cell presence. The glands remained in their healthy state, displaying no infiltration of inflammatory cells within the mucosal epithelial layer (Figure 7A). In contrast, the THP-withdrawn group displayed noticeable edemas in the mucosal villi, along with the infiltration of necrotic epithelial and inflammatory cells (Figure 7B). However, the co-treated group (THP + AEAP) showed no significant mucosal villi edema or necrosis, indicating the potential effectiveness of the combined treatment (Figure 7D). In the THP group post-treated with AEAP, mild edema was observed in the intestinal villi, accompanied by partial damage to these structures (Figure 7C).

## 4. Discussion

This study aimed to examine, for the first time, the intricate relationship between gut microbiota, oxidative stress, and prolonged THP administration, while investigating the potential therapeutic role of *A. pyrethrum* (AEAP) in alleviating these associated adverse withdrawal effects. Following the chronic administration of THP and its subsequent withdrawal, significant alterations in behavior were observed. These included an increased preference for the drug-paired chamber during the CPP test, indicating addictive tendencies. Concurrently, manifestations of anxiety and depressive-like behaviors emerged, along with a decrease in locomotor activity. Blood samples revealed elevated cortisol levels, signifying heightened stress during withdrawal. Furthermore, a reduction in both the density and diversity of the gut microbiome were observed, accompanied by evidence of elevated oxidative stress, as indicated by higher MDA and lower CAT and SOD levels. 

The misuse of medications for recreational or enhancement purposes, including anticholinergic agents like THP, has gained recognition in recent years [35]. These drugs, originally prescribed for addressing extrapyramidal symptoms in individuals undergoing neuroleptic therapy, have been increasingly associated with abuse and diversion. Among these, Benzhexol/THP has emerged as one of the most commonly abused anticholinergic agents, with documented psychotropic effects that include elevated states, euphoria, augmented energy, enhanced mood, amplified social interaction, and the potential for anticholinergic syndrome [6]. This syndrome can manifest with symptoms such as disorientation, hallucinations, paranoia, and confusion [36,37,38,39]. The abrupt cessation of anticholinergic medication has also been linked to withdrawal symptoms, such as heightened anxiety and insomnia [40]. To date, no prior investigation has scrutinized the withdrawal effects associated with THP. These pharmacological agents exert their impact by antagonizing the muscarinic acetylcholine receptor, typically recommended for their parasympatholytic influence [41]. The inhibitory outcomes on the dopaminergic neurons, which are conventionally balanced by the stimulatory actions of cholinergic neurons, can lead to a relative cholinergic surplus upon the blockade of dopamine receptors by antipsychotics [42]. This dynamic engenders extrapyramidal motor consequences, a balance that is mitigated through the administration of anticholinergic agents [43]. However, it is worth noting that anticholinergic drugs also manifest as robust, indirect dopamine agonists within the limbic system, contributing to their propensity for misuse [4]. Consequently, the chronic utilization of these substances has been linked to the emergence of tolerance and withdrawal phenomena, possibly stemming from the reinforcing impact of these abused agents on the mesolimbic dopaminergic system [6]. This system encompasses key regions, including the ventral tegmental area, the nucleus accumbens, and the prefrontal cortex [44]. Thus, the abrupt cessation of anticholinergic medication has been correlated with the onset of withdrawal syndrome, characterized by a spectrum of symptoms, including heightened anxiety, insomnia, and adverse effects such as memory impairment and alteration of the quality of life [45,46,47].

In this study, the chronic administration of THP induced oxidative stress, consistent with previous research [48] that has demonstrated that anticholinergic medications can lead to oxidative stress generation due to increased dopamine turnover [49]. The observed changes in intestinal tissue resulting from chronic THP administration may be linked to brain oxidative stress, as heightened central nervous system oxidative stress can indirectly affect the GM [50]. Additionally, the chronic effects of THP may contribute to these intestinal alterations, given one of its common side effects, which is constipation [1,51]. Chronic constipation is associated with large intestinal lesions [52] and a decreased abundance of beneficial bacteria, including *Alistipes*, *unclassified*_*o*__*Bacteroidales*, *Alloprevotella*, *Bilophila*, and *Anaerotruncus* [53]. Beneficial microbes operate by means of fermentation and the production of microbial metabolic products, contributing to the prevention of gut damage [54]. Although our study did not directly investigate THP withdrawal’s impact on the blood–brain barrier (BBB) or immune activation, our observations revealed perturbations in the GM during THP withdrawal. This alteration in the GM, as observed in the histopathological examinations, could potentially contribute to inflammation, indicative of immune system activation and the generation of ROS, all of which collectively led to oxidative stress. However, the administration of AEAP alongside THP, either preventively or during withdrawal, ameliorated the observed detrimental effects. This was accompanied by improvements in behavioral tests, cortisol levels, reduced oxidative stress, and the restoration of several GM species. This highlights the potential of AEAP to address THP-withdrawal effects. *A. pyrethrum* has demonstrated its potential for protecting against neurological disorders, such as Alzheimer’s, Parkinson’s, and epilepsy [55,56,57]. The neuroprotective properties of *A. pyrethrum* is attributed to its antioxidant and anti-inflammatory properties [21,58]. The use of AEAP has uncovered the existence of secondary metabolites (i.e., polyphenols, flavonoids, tannins, and alkaloids) that enhance γ-aminobutyric acid (GABA) transmission [21,22,23,58]. Furthermore, the phytochemical screenings of the *A. pyrethrum* roots revealed the main active constituent pellitorine—an N-isobutyl amide alkaloid—that contributes to AEAP’s therapeutic effects [22,59]. 

A bi-directional communication channel, known as the MGB, facilitates interactions between the gut and the brain. The precise mechanism through which the microbiome impacts behavioral changes remains uncertain [60,61,62,63], although numerous potential pathways are currently under investigation: (1) the stimulation of the vagus nerve, which connects the muscular and mucosal layers of the gastrointestinal tract to the brainstem, (2) the release of serotonin from enterochromaffin cells found in the gut’s epithelial lining, (3) microglia dysfunction, and (4) the direct transmission of chemical signals (i.e., toxins, short-chain fatty acids (SCFAs), and GABA) to the brain. These routes, either individually or in combination, contribute to the progression of the Aβ communication pathway from the gut to the brain. A growing body of evidence indicates that the GM and its byproducts, such as polyphenols, SCFAs, antioxidants, and vitamins, play a role in regulating numerous biosynthetic pathways. These pathways can have both positive and negative effects on the host system.

Emerging findings have indicated that adjusting the MGB axis can serve as an effective approach for addressing neurological disorders. Certain bacterial strains have demonstrated antioxidant capabilities, indicating their potential pro-/post-biotic and neuroprotective attributes [64]. Our previous study elucidated how AEAP influences the GM, promoting beneficial bacterial growth, such as *Lactobacillus* [23]. In fact, *Lactobacillus buchneri* KU200793, extracted from Korean fermented foods, exhibits elevated antioxidant capabilities. This strain has demonstrated the ability to shield SH-SY5Y cells from the negative impact of 1-methyl-4-phenylpyridinium (MPP+), shedding light on its potential probiotic and neuroprotective attributes [65]. In a similar vein, findings have indicated that exopolysaccharides obtained from *Lactobacillus* play a protective role for SH-SY5Y cells against apoptosis triggered by Aβ (1–42) [66]. Through scavenging ROS, probiotic bacteria might hinder the formation and aggregation of beta-amyloid fibrils, subsequently alleviating oxidative stress in the CNS [67]. In line with existing research, it has been found that probiotic supplements activate the sirtuin-1 protein deacetylase pathway, instigating antioxidative responses. Probiotic supplements, like SLAB51, composed of *Streptococcus thermophilus*, *Lactobacillus acidophilus*, *L. plantarum*, *L. paracasei*, *L. delbrueckii* subsp. *bulgaricus*, *L. brevis*, *Bifidobacterium longum*, *B. breve*, and *B. infantis,* have shown the ability to enhance antioxidant enzyme functionality, mitigating oxidative stress-related impairments. Notably, SLAB51 amplifies the functionality of antioxidant enzymes (i.e., catalase and guaiacol peroxidase), thereby mitigating oxidative stress-related impairments [68]. These consistent findings have been corroborated by human studies as well, further reinforcing the reliability of these observations. Gut bacteria, including *Lactobacilli* and *Bifidobacterium,* have the capacity to produce neurotransmitters (e.g., GABA) capable of regulating glucose homeostasis and modulating the host’s behavioral patterns. This has led to the restoration of metabolic and depressive-like abnormalities in mice [69,70].

Altogether, this study reveals that THP administration can lead to a withdrawal syndrome characterized by behavioral changes, biochemical impairments, increased oxidative stress markers, and GM dysbiosis. However, AEAP has demonstrated its potential to mitigate these adverse effects. The complex interplay among neurological outcomes, oxidative stress, and the GM involves numerous factors that warrant further investigation, including diet, lifestyle, and genetics, which were not extensively addressed in this study. This research opens the door to a promising area of study that may have a significant impact on our comprehension of the MGB connection and its role in neurological health.

## 5. Conclusions

The current study investigated, for the first time, the effects of prolonged THP administration on behavior, biochemical parameters, oxidative stress, and the gut microbiome. Chronic THP use was associated with adverse behavioral outcomes, including anxiety and depressive-like behaviors, as well as elevated cortisol levels indicative of increased stress. Furthermore, a reduction in the density and diversity of the gut microbiota was observed. However, the administration of AEAP as a co-and post-treatment emerged as a promising intervention. AEAP effectively mitigated THP’s adverse effects, improving behavioral outcomes and cortisol levels while restoring gut microbiome species. AEAP exhibited antioxidant properties, countering the oxidative damage caused by THP. Notably, post-treatment with AEAP demonstrated its potential to mitigate the withdrawal syndrome, while co-treatment effectively prevented its onset, highlighting the pivotal role of timing in intervention strategies. These findings offer new perspectives for substance use disorder management, emphasizing AEAP’s potential as a treatment option. Additional research is required to clarify its fundamental mechanisms and optimize AEAP’s clinical application, potentially revolutionizing the treatment of substance-related disorders.

## Data Availability

All data are contained in the article.

## Supplementary Materials

The following supporting information can be downloaded at: https://www.mdpi.com/article/10.3390/antiox13010026/s1, Figure S1: Graphic flowchart illustrating the experimental design, including the selection of rats, drug administration, behavioral tests, sample collection for gut microbiota, and oxidative stress analysis to investigate the modulation of the gut microbiome in trihexyphenidyl-induced behavioral and biochemical impairment in rats, and the potential of post-treatment with *A.pyrethrum* L. aqueous extract to mitigate the adverse effects; Table S1: Effects of trihexyphenidyl and/or aqueous extract of *Anacyclus pyrethrum* (AEAP) on the abundance of microbiota during the withdrawal phase.
