# Supplementary material for: Trihexyphenidyl Alters Its Host’s Metabolism, Neurobehavioral Patterns, and Gut Microbiome Feedback Loop—The Modulating Role of Anacyclus pyrethrum
Source: Antioxidants (Basel). 2023 Dec 21;13(1):26. doi: 10.3390/antiox13010026 (PMC10812446; doi:10.3390/antiox13010026)
Supplement: Supplementary file 1 [file antioxidants-13-00026-s001.zip › antioxidants-2725501-supplementary.pdf]

Supplementary data

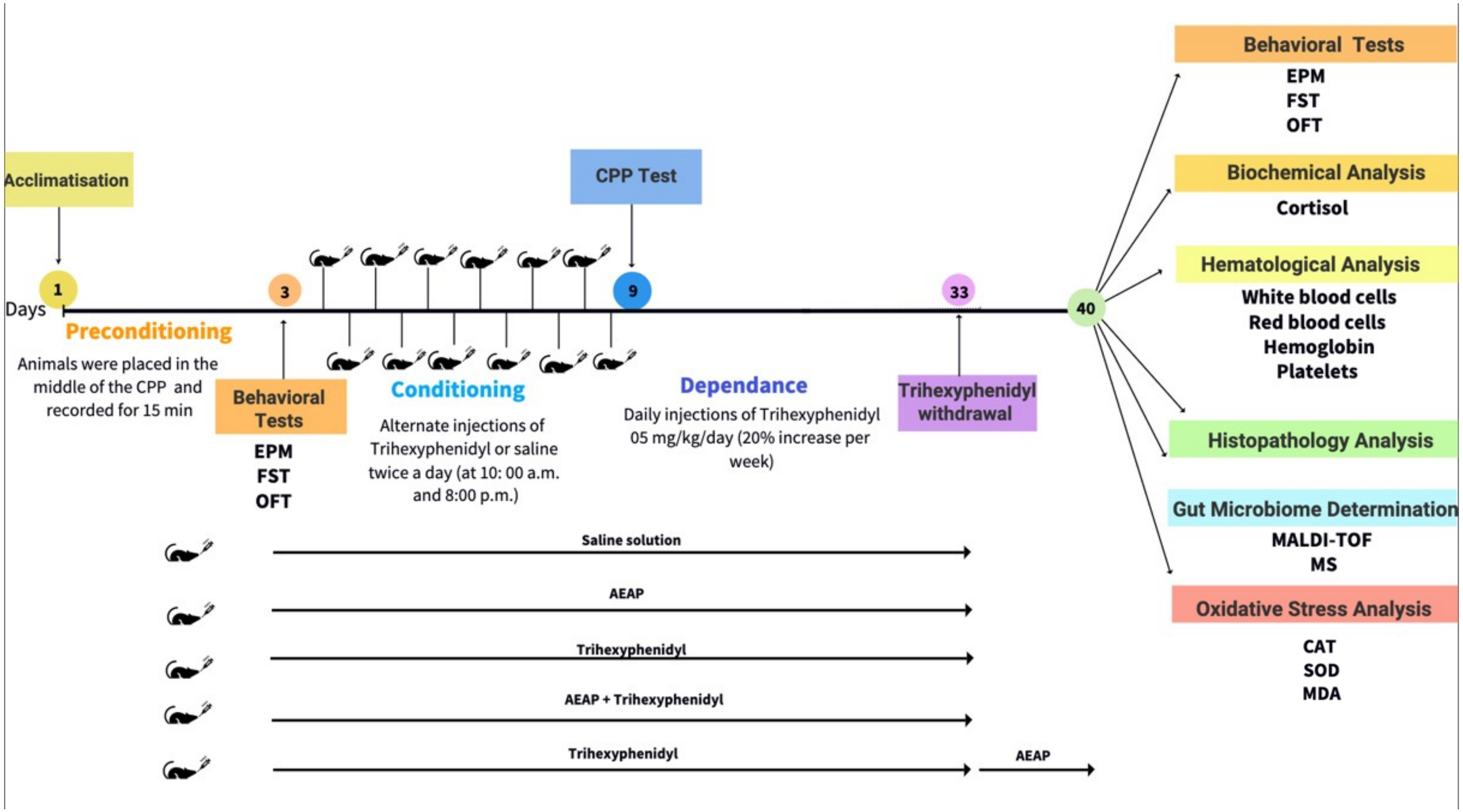

Figure S1. Graphical flowchart illustrating the experimental design, including the selection of rats, drug administration, behavioral tests, and sample collection for gut microbiota and oxidative stress analysis to investigate the modulation of the gut microbiome in Trihexyphenidyl induced behavioral and biochemical impairment in rats and the potential of post-treatment with *A.pyrethrum* L. aqueous extract to mitigate adverse effects.

Table S1. Effects of Trihexyphenidyl and/or aqueous extract of *Anacyclus pyrethrum* (AEAP) on the abundance of the microbiota during the withdrawal phase.

	CFU x10⁶/ mL			
	Control	THP withdrawn	THP+AEAP post-treatment	THP+AEAP co- treatment
**Phylum**				
Firmicutes	12.78	1.91	2.99	15.00
Proteobacteria	1.91	0.39	0.67	2.00
**Orders**				
Lactobacillales	12.78	1.84	2.99	14.00
Bacillales	0.00	0,07	0.00	1.00
Pasteurellales	1.91	0.00	0.59	1.00
Enterobacterales	0.01	0,39	0.08	1.00
**Family**				
Lactobacillaceae	12.20	1.84	2.92	14.00
Staphylococcaceae	0.00	0.07	0.00	1.00
Streptococcaceae	0.58	0.00	0.00	0.00
Pasteurellacea	1,91	0.00	0.004	1.00
Enterobacteriaceae	0.01	0.39	0,08	1.00
**Species**				
Ligilactobacillus faecis	9.26	1.84	1.46	14.00
Limosilactobacillus reutri	2.94	0.00	1.46	0.00
Staphylococcus hominis	0.00	0.07	0.00	0,00
Streptococcus anginosus	0.29	0.00	0.00	0,00
Streptococcus hyointestinalis	0.29	0.00	0.07	0,00
Haemophilus haemolyticus	0.29	0.00	0.04	1.00
Rodentibacter ratti	1.62	0.00	0.55	1.00
Escherichia Coli	0.01	0.39	0.08	1.00
